# A Sensor-Based Method for Diagnostics of Machine Tool Linear Axes

**Published:** 2015

**Authors:** Gregory W. Vogl, Brian A. Weiss, M. Alkan Donmez

**Affiliations:** 1 National Institute of Standards and Technology (NIST), Gaithersburg, Maryland, 20899, USA

## Abstract

A linear axis is a vital subsystem of machine tools, which are vital systems within many manufacturing operations. When installed and operating within a manufacturing facility, a machine tool needs to stay in good condition for parts production. All machine tools degrade during operations, yet knowledge of that degradation is illusive; specifically, accurately detecting degradation of linear axes is a manual and time-consuming process. Thus, manufacturers need automated and efficient methods to diagnose the condition of their machine tool linear axes without disruptions to production. The Prognostics and Health Management for Smart Manufacturing Systems (PHM4SMS) project at the National Institute of Standards and Technology (NIST) developed a sensor-based method to quickly estimate the performance degradation of linear axes. The multi-sensor-based method uses data collected from a ‘sensor box’ to identify changes in linear and angular errors due to axis degradation; the sensor box contains inclinometers, accelerometers, and rate gyroscopes to capture this data. The sensors are expected to be cost effective with respect to savings in production losses and scrapped parts for a machine tool. Numerical simulations, based on sensor bandwidth and noise specifications, show that changes in straightness and angular errors could be known with acceptable test uncertainty ratios. If a sensor box resides on a machine tool and data is collected periodically, then the degradation of the linear axes can be determined and used for diagnostics and prognostics to help optimize maintenance, production schedules, and ultimately part quality.

## 1 Introduction

Linear axes are used to move components of machine tools that carry the cutting tool and workpiece to their desired positions for parts production (Altintas, Verl, Brecher, Uriarte & Pritschow, 2011). Essentially, a linear axis moves along a nominally linear path and is a vital subsystem of computer numerical control (CNC) machine tools. Because a typical 3-axis machine tool has three linear axes, their positional accuracies directly impact load capacity, quality, and efficiency of manufacturing processes.

As a machine tool is utilized for parts production, emerging faults lead to performance degradation, which lowers control precision and accuracy (Li, Wang, Lin & Shi, 2014). Typical faults within feed systems are due to pitting, wear, corrosion, cracks, and backlash (Zhou, Mei, Zhang, Jiang & Sun, 2009). As degradation increases, tool-to-workpiece errors become more likely, and eventually, linear axes of CNC machines may undergo significant wear that results in a failure and/or a loss of production quality (Uhlmann, Geisert & Hohwieler, 2008). Occurrences of faults and failures are becoming more common as higher levels of automation and productivity within manufacturing result in greater wear on machine components. Machine tool faults account for yearly economic losses of tens of billions of US dollars (Shi, Guo, Song & Yan, 2012). Thus, machine tools must be maintained and available for cost-effective production (Verl, Heisel, Walther & Maier, 2009).

Yet knowledge of degradation is illusive; accurately detecting degradation of linear axes is a manual, time-consuming, and potentially cost-prohibitive process. While direct methods for machine tool calibration are well-established (International Organization for Standardization, 2012) and reliable for position-dependent error quantification, measurements for these methods typically halt production and take “a long time” (Khan & Chen, 2009). The “extensive experimental and analytical efforts” for conventional sequential error measurement methods is usually time-consuming and requires expensive equipment, hindering widespread commercial adoption (Ouafi & Barka, 2013). Because degradation differs along a linear axis and the wear changes with production time (Uhlmann et al., 2008), the particular condition of an axis is usually unknown. The varying loads, hardness, and surface friction of guides affect their performance, so prediction of remaining useful life (RUL) of linear axis guideways may be difficult (Huang, Gao, Xu, Wu, Zhao & Guo, 2010).

Manufacturers need automated and efficient methods for continual diagnosis of the condition of machine tool linear axes without disruptions to production. This need is consistent with a European roadmap that identified three main key enabling technologies (KETs) for the future of sensor technology in manufacturing: new sensors and sensor systems, advanced sensor signal data processing, and intelligent sensor monitoring (Teti, Jemielniak, O’Donnell & Dornfeld, 2010). An online, condition monitoring system for linear axes is needed to help achieve the roadmap goals: decreased machine downtime, higher productivity, higher product quality, and enhanced knowledge about manufacturing processes (Teti et al., 2010).

Efforts to monitor the condition of linear axes components have utilized various sensors:

- Built-in linear and motor encoders (Plapper & Weck, 2001, Zhou, Tao, Mei, Jiang & Sun, 2011, Zhou, Xu, Liu & Zhang, 2014) with laser interferometer (Verl et al., 2009)
- Motor torque via current sensors (Li et al., 2014, Uhlmann et al., 2008, Zhou et al., 2009), accelerometers (Feng & Pan, 2012, Huang et al., 2010, Liao & Lee, 2009)
- Accelerometers, thermocouples, and analog controller outputs (torque, speed, and encoder position) (Liao & Pavel, 2012)
- Hall effect sensors (Garinei & Marsili, 2012)
- Piezoresistive thin films (Biehl, Staufenbiel, Recknagel, Denkena & Bertram, 2012, Möhring & Bertram, 2012)
- Piezoelectric ceramics (Ehrmann & Herder, 2013).

These attempts at condition monitoring of linear axes were limited in success, largely because both external sensors and built-in sensors have limitations. Built-in position sensors are usually highly accurate (Zhou et al., 2011), yet controller signals have problems such as low sample rate, limited sensitivity due to sensors being far from monitored components, and unwanted influences from multiple sources (Plapper & Weck, 2001). On the other hand, external sensors can be more direct and physically sensitive, but high costs and required bandwidths have impeded their application for online monitoring of linear axes (Zhou et al., 2009). Adding sensors to machine tools can also be very time-consuming with respect to setup, integration, and data communication.

In this paper, a new sensor-based method for diagnostics of machine tool linear axes is presented. The Prognostics and Health Management for Smart Manufacturing Systems (PHM4SMS) project at the National Institute of Standards and Technology (NIST) developed a sensor-based method to quickly estimate the performance degradation of linear axes. External sensors are used for high-bandwidth direct or indirect measurements of changes in linear axis errors. The sensors are contained within a ‘sensor box’ for ease of installation and periodic use on a machine tool for data collection and analysis, e.g., within 5 min. The diagnostics and prognostics of the linear axes can be used to help optimize maintenance, production schedules, and ultimately part quality. The cost-effective sensors are expected to be an overall net positive when factoring in the expected savings in production losses and scrapped parts for a machine tool.

## 2. Sensor Box Concept for Metrology

The goal of the new sensor-based method is to enable efficient monitoring of the change in positioning errors, and hence the change in tool-to-workpiece positioning performance, due to degradation of linear axes. This section outlines these errors, the concept of the sensor-based methodology, and the needed uncertainties of the method.

### 2.1. Straightness and Angular Errors

**Figure 1:**
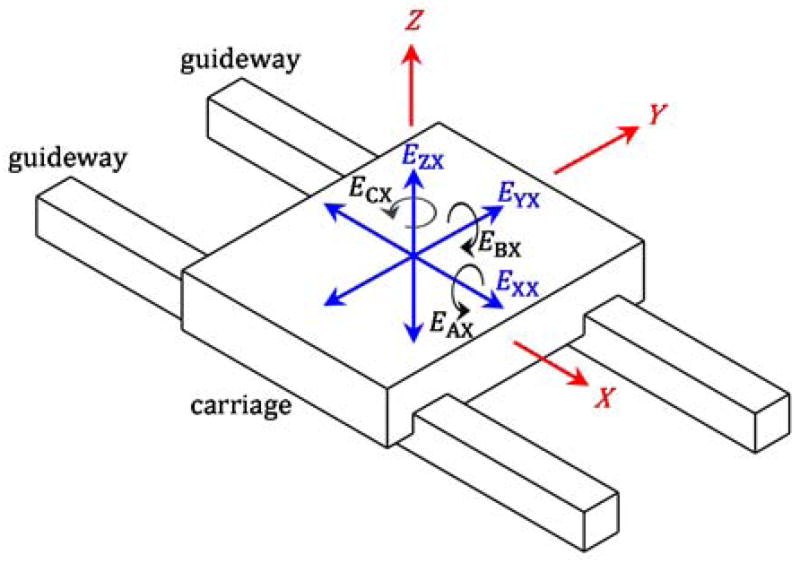
Translational and angular errors of a component commanded to move along a (nominal) straight-line trajectory parallel to the X-axis.

Even without degradation, the carriage of a linear axis translates and rotates due to imperfections as the carriage moves along the guideways of the linear axis. Figure 1 shows these six errors that change with axis degradation. As the carriage is positioned along the X axis, it encounters three translational errors from its nominal path: one linear displacement error (*E*_XX_) in the X-axis direction and two straightness errors (*E*_YZ_ and *E*_ZX_) in the Y- and Z-axis directions. The carriage also experiences three angular errors (*E*_AX_, *E*_BX_, and *E*_CX_) about the X-, Y-, and Z-axes.

A typical machine tool has three linear axes, which means that a total of 18 (= 6 × 3) translational and angular errors exist. These errors are major contributors to the position-dependent tool-to-workpiece errors.

### 2.2. Sensor Box Concept

**Figure 2:**
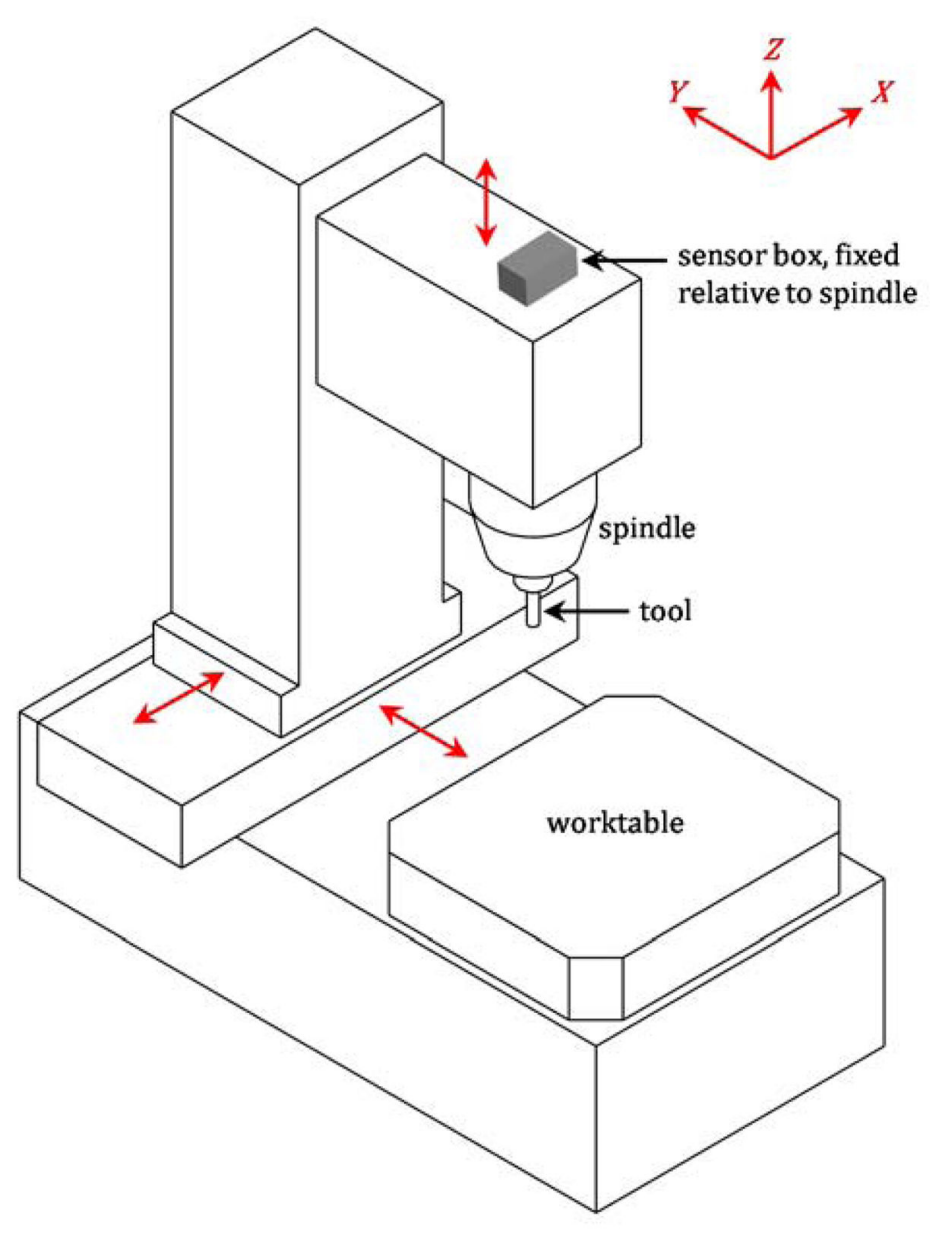
Schematic of sensor box on machine tool for metrology of linear axis degradation.

Sensors can be used to measure changes in the straightness and angular errors due to degradation. Figure 2 shows a sensor box on a typical 3-axis machine tool with ‘stacked’ linear axes; the Z axis is on the X axis, which is on the Y axis. The sensor box is attached to the Z-axis slide, so that if any axis is moved, the sensor box moves and will detect motion. Accelerometers are used to detect translational errors, and inclinometers and rate gyroscopes are used to detect angular errors. Some properties of these sensors are outlined in Table 1.

Once collected, the sensor data is processed to yield the straightness and angular errors. Specifically, rate gyroscope signals are integrated once to yield angular changes, and accelerometer signals are integrated twice to yield translational errors. Inclinometers may be used for direct measurement of angle from 0 Hz to about 2 Hz, as seen in Table 1. The reason for two types of angular sensors is that the inclinometer may measure low-frequency angular error terms with greater accuracy than the rate gyroscope.

Degradation may be tracked periodically by data collection during a fixed-cycle test (Garinei & Marsili, 2012, Huang et al., 2010, Liao & Lee, 2009, Verl et al., 2009, Zhou et al., 2009, Zhou et al., 2011). During a fixed-cycle test, the machine tool axes are commanded to move via the same program (the fixed cycle) with the machine tool initially in the same state (temperature, etc.) and undergoing the same nominal loads (cutting forces, if cutting occurs). The collected data is then processed, and the fixed-cycle results are compared to the previous results to determine the changes in straightness and angular errors. The deviations from one test to another are due to degradation, typically due to mechanical wear.

For the machine tool configuration highlighted in Figure 2, changes in the positioning errors could be estimated by using the data from the sensor box and the box’s position relative to the tool tip. Therefore, the sensor box is focused on tracking the effects of degradation of each linear axis on the machining performance. For 4- or 5-axis machine tools with rotary axes, the rotary axes would be held fixed during motion of the linear axes. Also, for a different machine configuration without 3-axis stacking, an additional sensor box on the worktable would be necessary.

Details of the fixed-cycle test and data processing for the determination of error changes will be described in later sections.

### 2.3. Tolerances for Errors

The sensor-based method depends on the available sensors, whose selection depends on the magnitude of errors to be detected and the accuracy with which they need to be identified. Small levels of degradation of linear axes are expected and allowed, but there are limits specified for axis errors. ISO 10791-2 (International Organization for Standardization, 2001) specifies the tolerances for linear axis errors of vertical machining centers. As shown in Table 2, the acceptable straightness error is limited to 20 μm and the acceptable angular error is limited to 60 μrad.

The measurement uncertainties must be less than the respective specified tolerances to measure the errors. The test uncertainty ratio (TUR), which is the ratio of the tolerance to the uncertainty of the measurement, should be sufficiently large. Typically, a TUR of at least 4:1 is recommended; the larger, the better for a measurement system. For the measurement system to be created, we will accept a TUR of at least 4:1 based on design constraints such as sensor cost and size. Thus, we will accept straightness and angular error measurement uncertainties of 5 μm and 15 μrad, respectively, based on the tolerances outlined in Table 2.

## 3. Sensor-based Methodology

A sensor-based method was developed to satisfy the TUR constraint of 4:1 and a total cost of about US$5000 for sensors. This section summarizes the sensor box, the fixed-cycle test, and the sensor-based methodology for determination of changes in straightness and angular errors.

### 3.1. Sensor Box

**Figure 3:**
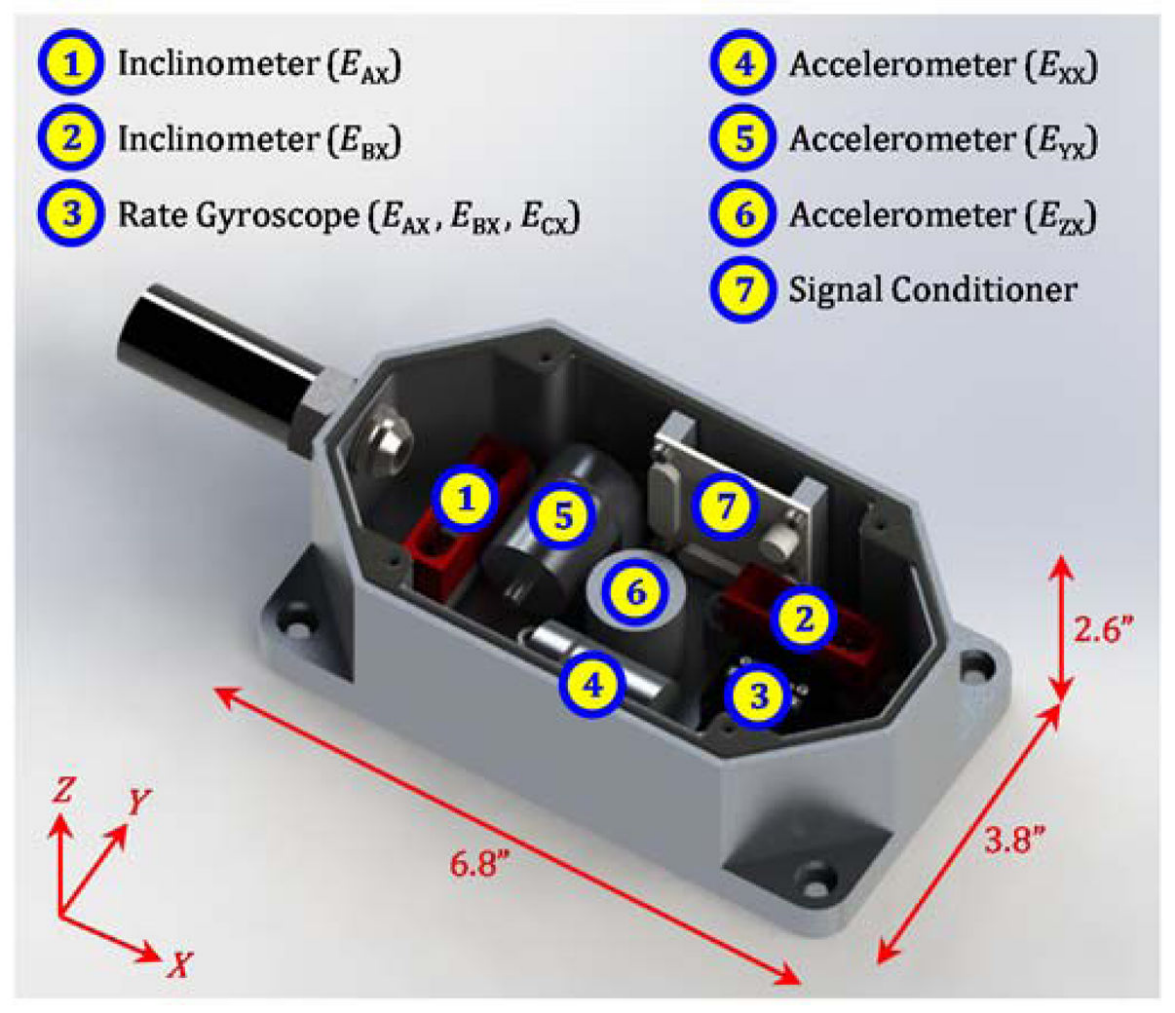
Rendered image of sensor box with sensors.

Figure 3 presents the sensor box, which is composed of two inclinometers, one tri-axial rate gyroscope (three rate gyroscopes), and three accelerometers. Each sensor detects a component of the translational or angular errors seen in Figure 1. The relationships of the sensors to these error components are noted in Figure 3.

The sensor box top is not shown in Figure 3, so the sensors and their placement can be seen. When the sensor box top is attached, a rubber seal between the box top and base ensure that the sensors are sealed for protection from machine tool environments (including fluids, metal chips, etc.).

### 3.2. Fixed-Cycle Test

Table 3 summarizes the fixed-cycle test for degradation metrology. For the fixed-cycle test, each of the axes is operated sequentially to move over its entire travel range at three constant speeds typical of linear axes: ‘Slow’ axis speed = 0.02 m/s (50 s to travel 1 m), ‘Moderate’ axis speed = 0.1 m/s (10 s to travel 1 m), and ‘Fast’ axis speed = 0.5 m/s (2 s to travel 1 m). Different axis speeds are used to account for the various sensor bandwidths and noise properties seen in Table 1, in order to minimize the measurement uncertainties of the estimated translational and angular errors. For example, the inclinometer requires a ‘slow’ speed due to its bandwidth of 2 Hz, while the accelerometer requires faster speeds to sense low spatial frequency motions due to its low cutoff frequency of 0.02 Hz. If data is collected for only the forward motion of each axis of a 3-axis machine tool, then the data collection time totals about 3 min (= 3 × (50 s + 10 s + 2 s)).

**Figure 4:**
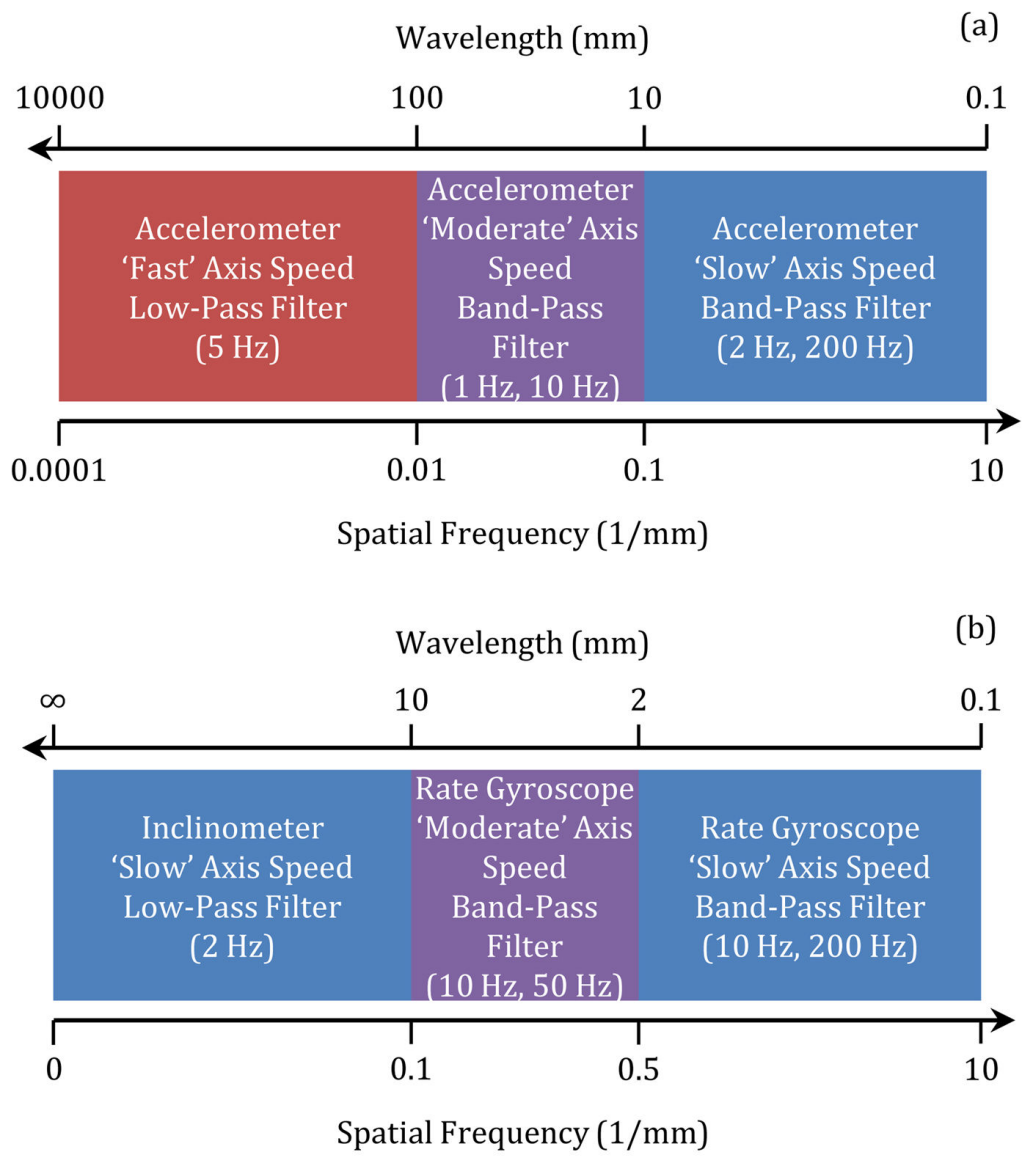
Fixed-cycle test data analysis for (a) straightness errors and (b) angular errors.

Sensor data is collected, integrated (as needed), filtered, and processed to yield the error components noted in Figure 3. These ‘data fusion’ processes are based on the fact that signals generated by the same geometric errors can be decomposed into various frequency components via filtering and then added together to yield the original errors. As seen in Figure 4, each filtered sensor signal yields a portion of the same geometric error over different neighboring spatial frequency ranges. Because these frequency ranges border each other, the error components add together to result in the originating geometric errors with wavelengths down to 0.1 mm.

Specifically, the rate gyroscope signal is filtered with 2-pole Butterworth filters, integrated, and then summed to the raw inclinometer signal to yield the angular errors. The only exception is for the Z axis, which does not have an inclinometer (as indicated in Figure 3), so the rate gyroscope is used alone to yield *E*_CX_. Also, the filtered outputs from the accelerometer signals collected at different speeds can be summed, with the resultant acceleration integrated twice to yield straightness errors. The sensors must have relatively low noise in order to minimize drift, especially for the straightness errors based on double integration.

## 4 Sensor-Based Method Uncertainty

Uncertainty is inherent with physical measurements, and the sensor-based method is no exception. Various sources of uncertainty exist, including sensor misalignment, calibration, and nonlinearity, as well as modal vibrations that could influence the signals. However, this section focuses on the expected main sources of uncertainty to the straightness and angular error estimates: sensor noise and the data fusion process described in Section 3.2.

### 4.1. Uncertainty Contributions from Sensor Noise

Each sensor has specified noise levels that influence the recorded sensor values. When processed according to Figure 4, sensor noise contributes uncertainty to the straightness and angular errors. Table 4 and Table 5 summarize these uncertainty contributions, determined from numerical simulations based on product specifications (e.g., see Table 1) in which 500 trials were used for statistical purposes. For example, the 10-second long (for the ‘moderate’ speed) simulated noise signal for the rate gyroscope was sampled at 25.6 kHz, a possible experimental sampling rate. The root mean square (RMS) of the spectral density of the white noise was scaled to match the RMS of the spectral density (0.002 °/√Hz) of the sensor, as specified in the product datasheet. The simulated noise was band-pass filtered with 2-pole Butterworth filters with a lower cutoff frequency of 10 Hz and an upper cutoff frequency of 50 Hz. Next, the filtered angular velocity was integrated to determine the angular displacement noise. Out of 500 trials, the mean was negligible, so the standard uncertainty is approximately the RMS deviation. The largest angular displacement was shown to be 6.2 μrad, and the RMS angular displacement was about 1.2 μrad for all trials, as seen in Table 5. Similarly, simulated acceleration signals were filtered and double-integrated to yield the translational displacement noises seen in Table 4.

The combined standard uncertainty over the full spatial spectrum due to sensor noise is equal to the square root of the sum of individual standard uncertainties listed in Table 4 or Table 5. Therefore, the combined standard uncertainty of the straightness error is 0.064 μm (= [(0.015 μm)^2^ + (0.029 μm)^2^ + (0.055 μm)^2^]^1/2^) and the combined standard uncertainty of the angular error is 2.3 μrad (= [(1.4 μrad)^2^ + (1.2 μrad)^2^ + (1.3 μrad)^2^]^1/2^). The combined expanded uncertainties for a coverage factor of *k* = 5, similar to those in Table 4 and Table 5, are 0.32 μm and 11.3 μrad, respectively, for straightness and angular errors.

The uncertainty evaluations are based on Monte Carlo propagation of the contributions from the recognized sources of uncertainty. The resulting expanded uncertainties are the half-widths of coverage intervals that include 99.8 % of the Monte Carlo sample of values of the measurand. The corresponding coverage factor was obtained as the ratio between the expanded uncertainty and the standard uncertainty. The unusually large size of this factor (*k* = 5) is attributable to the fact that the probability distribution of the measurand is markedly non-Gaussian.

Based on the tolerances of 20 μm and 60 μrad in Table 2, the TUR for noise-related straightness error is about 63:1 (= 20 μm/0.32 μm) and the TUR for noise-related angular error is about 5:1 (= 60 μrad/11.3 μrad). Because the TURs related to sensor noise satisfy the given constraint of 4:1, the sensors are acceptable.

### 4.2. Uncertainties of Sensor-Based Method

However, uncertainties of the straightness and angular errors are due to not only sensor noise, but also due to the data fusion process described in Section 3.2. Thus, the complete processes outlined in Figure 4 (with sensor noise included) were simulated for different randomly-generated straightness errors and angular errors within the tolerances (20 μm and 60 μrad) seen in Table 2. For any trial, the errors are generated in a process similar to a random-walk. Once generated, the simulated straightness and angular errors are considered to be the ‘reference’ errors, i.e., the ‘true’ errors, which can be compared to the ‘estimated’ errors resulting from the processes described in Section 3.2.

**Figure 5:**
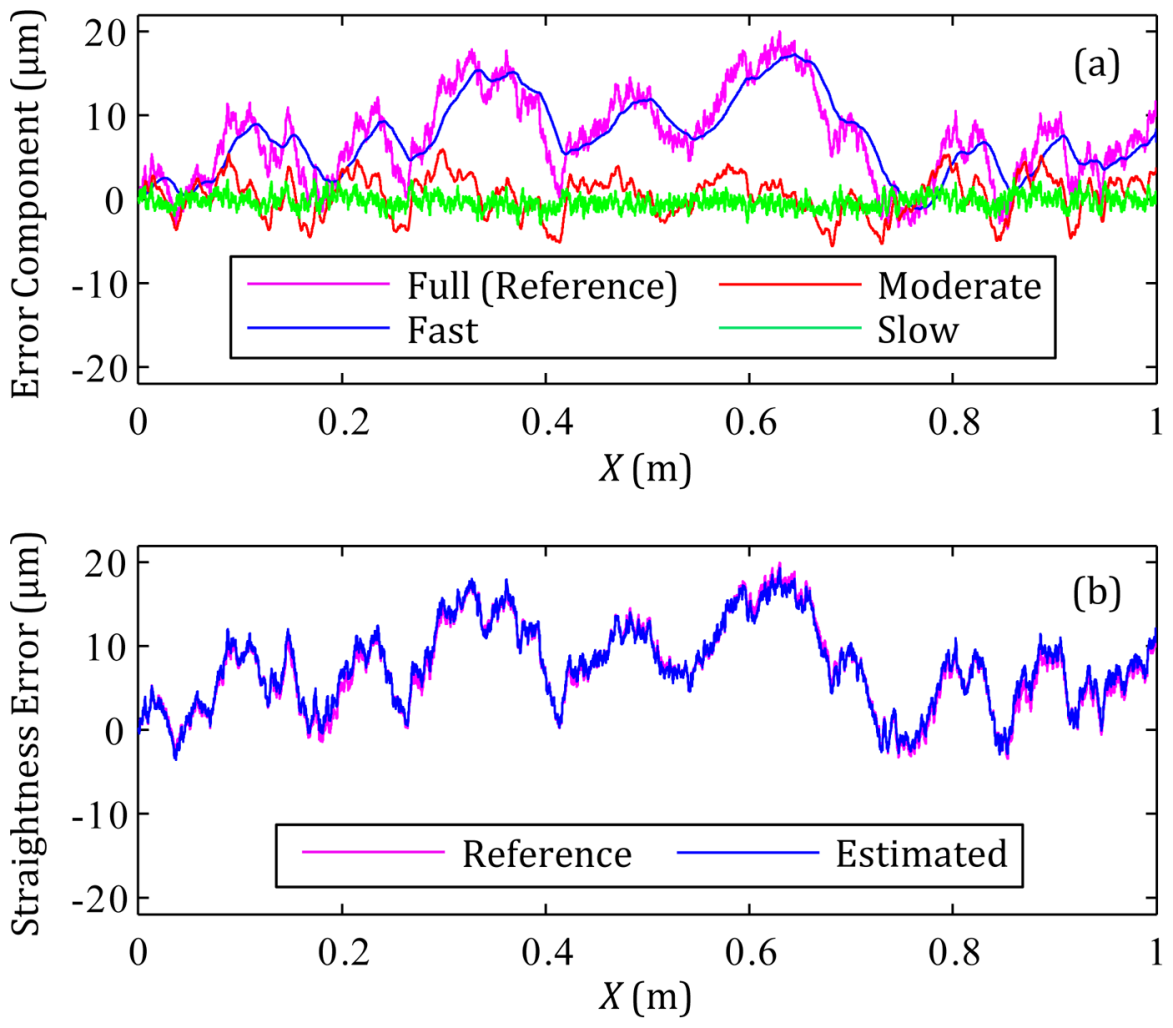
Example estimation of straightness error: (a) Straightness error component for each axis feed rate and (b) reference straightness error versus the estimated straightness error.

Figure 5(a) shows the three individual components of straightness error for one simulation that are summed to yield the estimated straightness in Figure 5(b). The ‘Fast’ axis-speed component is composed of the lowest frequency terms, while the ‘Slow’ axis-speed component is composed of the highest frequency terms.

For 100 simulations with different randomly-generated straightness errors (the ‘reference’ errors), the difference between the reference and estimated straightness errors was within ± 5.6 μm, and the RMS of the difference over the entire axis travel was typically around 0.97 μm.

**Figure 6:**
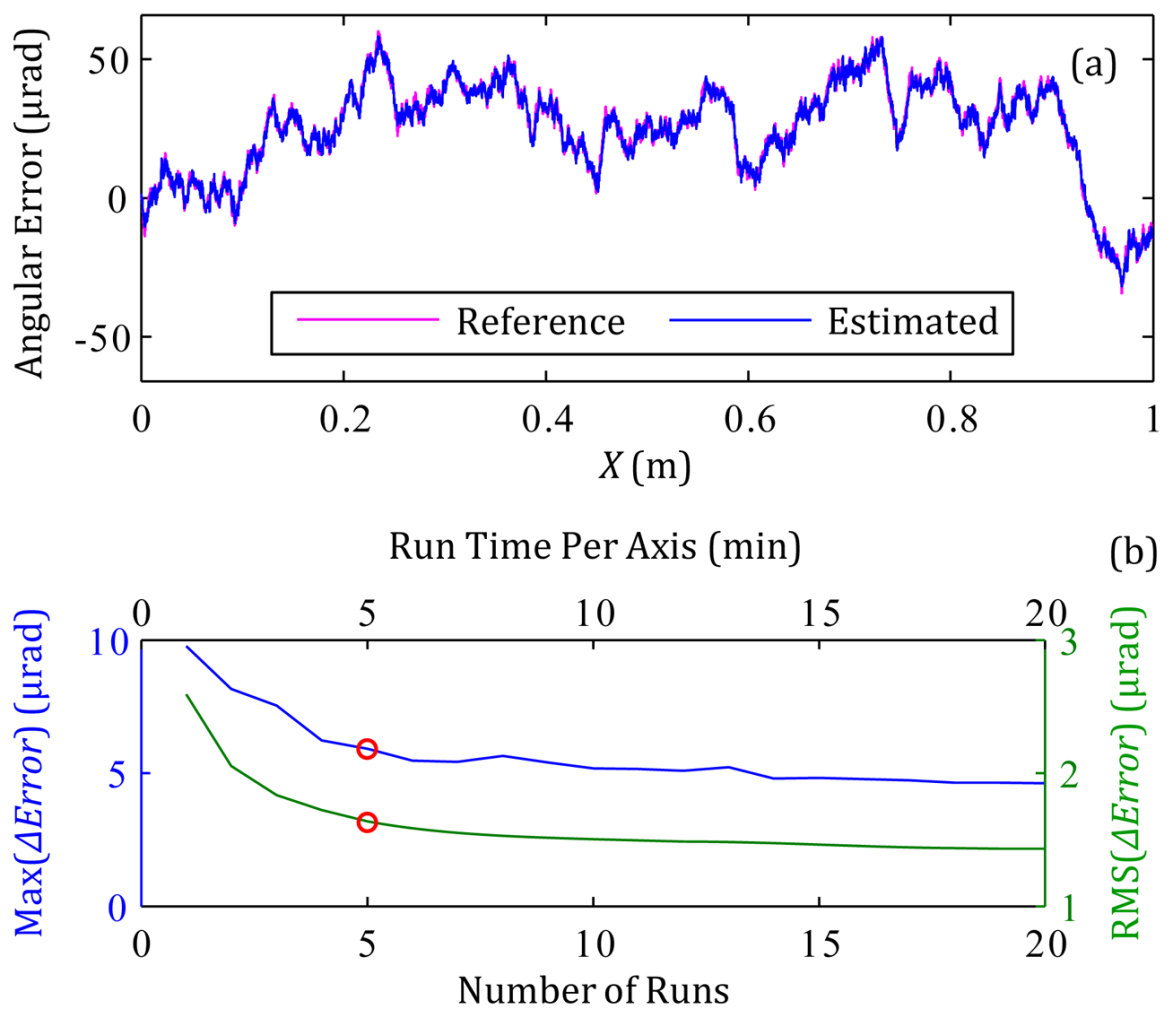
(a) the average estimated angular error for 5 runs and (b) the maximum and RMS values of *ΔError* versus the number of runs.

The estimation of the straightness and angular errors could be improved with averaging the results of multiple runs for data collection. For one case, Figure 6(a) shows the estimated angular error resulting from the use of 5 runs for averaging, and Figure 6(b) shows how the maximum and RMS values of *ΔError*(= estimated angular error – reference angular error) change with the number of runs used for averaging. Figure 6(b) shows that the maximum difference and RMS values approach 4.6 μrad and 1.4 μrad, respectively, as the number of runs for averaging increases. Both values do not approach zero as the number of runs increases towards infinity, because the process of Figure 4(b) is not perfect with respect to filtering or data fusion.

Table 6 shows the uncertainties of the sensor-based method for both straightness and angular error estimations with various numbers of runs for averaging (1, 5, or 10).

Based on Figure 6(b) and Table 6, the number of runs should be no more than 5 runs (or 15 minutes of total data acquisition time for three axes), because more than 5 runs is time-consuming with minimal gain in accuracy. This result is consistent with, and helps to support, international machining standards that utilize 5 runs in any direction (positive or negative) for averaging purposes, e.g., Section A.3.1 in ISO 230-2:2014 (International Organization for Standardization, 2014).

### 4.3. Method Limitations

Based on Table 2 and Table 6, the TUR for straightness error is about 5:1 (= 20 μm/4.1 μm) and the TUR for angular error is about 7:1 (= 60 μrad/9.0 μrad) for 5 runs used for averaging. Both TURs satisfy the given constraint of 4:1, so the process described in Section 3.2 is acceptable.

**Figure 7:**
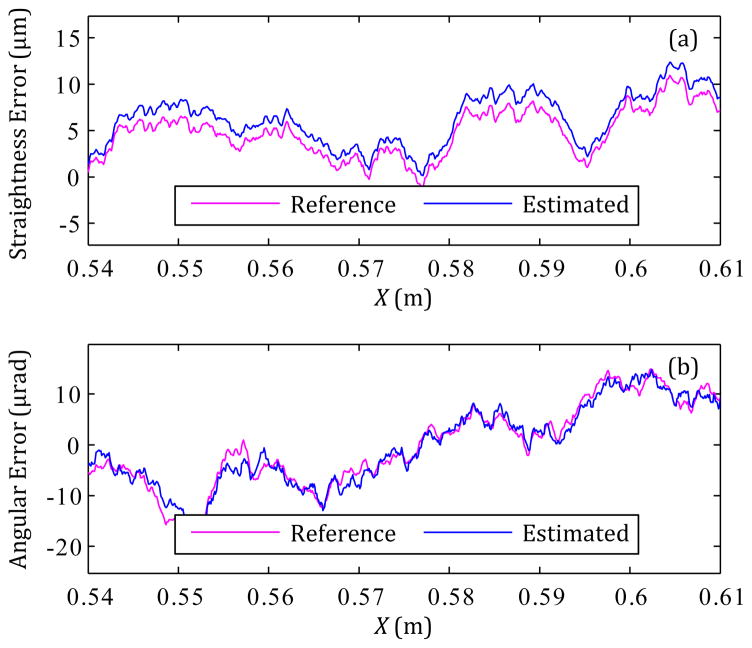
Typical section of (a) estimated straightness error and (b) estimated angular error, based on 5 runs used for averaging.

Nonetheless, the method is limited because neither the sensors nor the data fusion process described in Section 3.2 are perfect. Comparison of the straightness error uncertainties due to either noise (see Table 4) or the entire method (see Table 6) shows that the latter is dominant; the accelerometer noise is a minor contributor to measurement uncertainty. In fact, the major source of straightness error uncertainty is the limited sensor bandwidth; the lower cutoff frequency of the accelerometer is not 0 Hz but rather 0.02 Hz (3 dB). Hence, the spatial frequency of Figure 4(a) does not reach down to 0 mm^−1^. Figure 7(a) shows how the main local difference between the reference and estimated straightness errors is basically a low-frequency shift.

In contrast, Table 5 and Table 6 show how the angular sensor noise, especially that of the rate gyroscope, is a major contributor to the angular error uncertainty. Consequently, the main local difference between the reference and estimated angular errors is higher-frequency in nature, as seen in Figure 7(b).

## 5. Implementation of Sensor-Based Method

The new sensor-based methodology for diagnostics of machine tool linear axes must be tested, validated, and verified experimentally. This section outlines the means for testing the accuracy of the sensor-based method for the detection of straightness and angular errors.

### 5.1. Linear Axis Testbed

**Figure 8:**
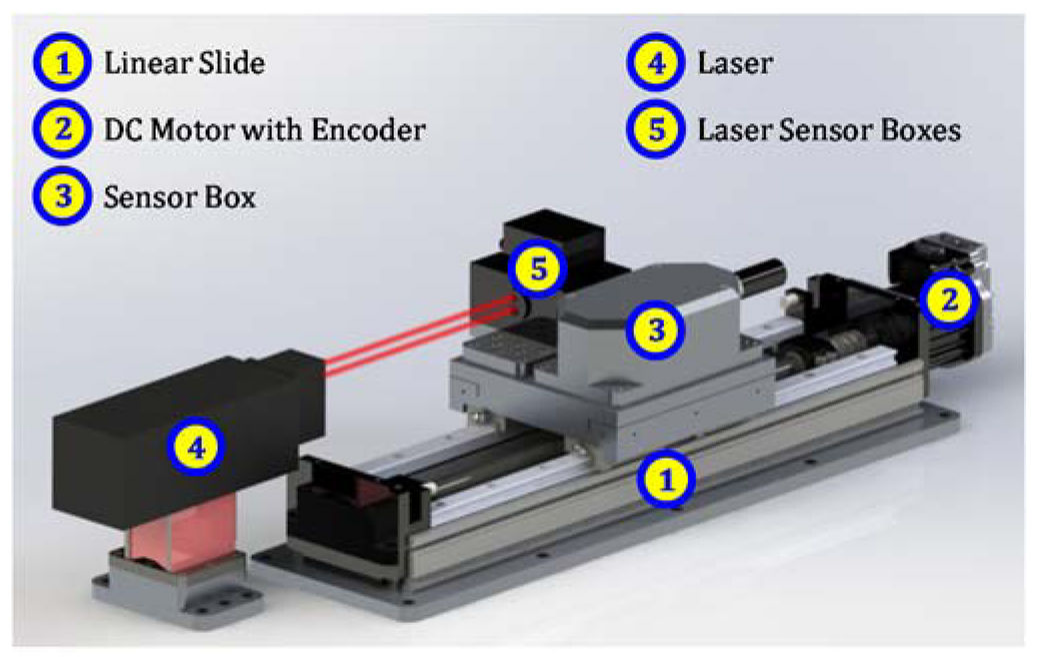
Rendered image of linear axis testbed for testing of sensor-based methodology.

A linear axis testbed was designed for testing the sensor-based method. As seen in Figure 8, the testbed is composed of a linear slide with a travel length of 300 mm. The linear slide is driven by a direct current (DC) motor with a rotary encoder attached to the motor shaft for motion control. Position is detected with a resolution of about 5 μm, which is much smaller than the 0.1 mm resolution of the method (see Table 3 or Figure 4) to enable repeatable test results.

Sensor boxes move with the carriage: the ‘sensor box’ for the new method and other boxes for a commercial laser-based system. The main laser sensor box contains optical technology to achieve a straightness error uncertainty of ±0.7 μm and an angular error uncertainty of ±3.0 μrad for 300 mm of travel. Due to its accuracy and precision, the laser-based system is used for validation and verification of the sensor-based method results.

### 5.2. Experimental Method

The sensor-based method must be tested to determine its efficacy in measuring changes, due to degradation, in straightness and angular errors of linear axes. One possible approach to induce degradation signals is to physically wear the linear slide, shown in Figure 8. However, such an approach is potentially time-consuming, expensive, and not repeatable due to unpredictable wear patterns.

**Figure 9:**
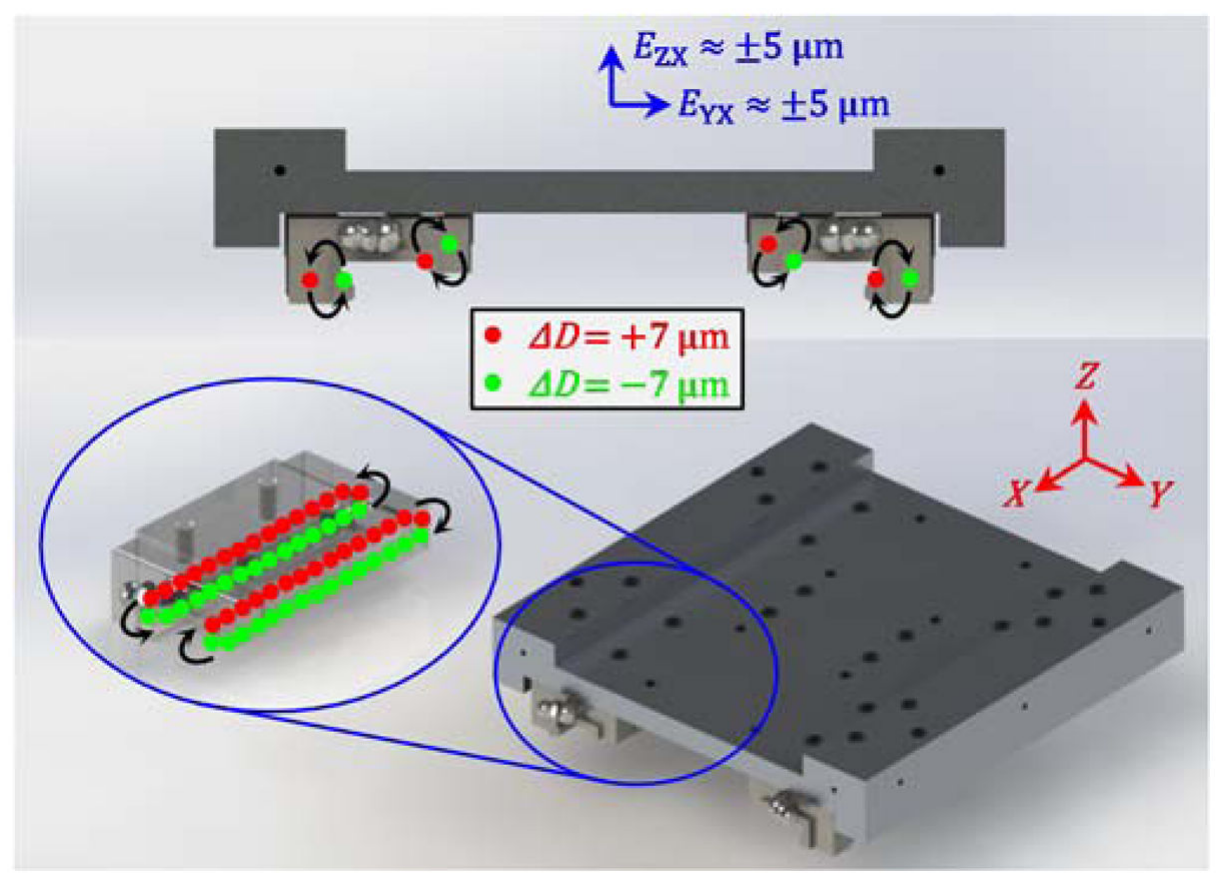
Example of experimental simulation of linear axis degradation via changes (*ΔD*) to ball diameters.

In contrast, we choose to experimentally simulate degradation by replacing the default ball bearings with those of different diameters, as illustrated in Figure 9. The linear slide contains four ‘blocks’ or ‘trucks’, each with recirculating balls that contact the rails to constrain the carriage along its nominally linear path. Initially, every ball has the same nominal diameter of approximately 3.972 mm. These default balls can be replaced with balls of smaller or greater diameter to induce straightness and angular error changes of the carriage. The change (*ΔD*) of ball diameter is experimentally simple, quick, inexpensive, and repeatable.

For example, Figure 9 shows how half of the balls can be replaced with balls that are 7 μm larger (*ΔD* = 7 μm) and the other half can be replaced with balls that are 7 μm smaller (*ΔD* =− 7 μm). The net result is that the straightness errors, *E*_YX_ and *E*_ZX_, will transition between about 5 μm and −5 μm as the carriage moves along the linear axis, for straightness error changes of about 10 μm. A variety of other ball configurations can cause translational or rotational changes of 20 μm or 60 μrad, respectively, which are the maximum acceptable errors according to Table 2. Therefore, patterns of balls of various diameters can be used to experimentally simulate error changes due to wear.

## 6. Conclusions

Manufacturers need quick and automated methods for continual diagnosis of machine tool linear axes without disruptions to production. Towards this end, a new sensor-based method was developed for linear axis diagnostics. The method uses a sensor box composed of inclinometers, accelerometers, and rate gyroscopes for high-bandwidth direct or indirect measurements of straightness and angular errors. When filtered and fused, the data yields seamless errors with wavelengths down to 0.1 mm. Simulations revealed that the multi-sensor-based method is capable of achieving test uncertainty ratios (TURs) of at least 4:1.

The sensor-based method must be validated and verified. Thus, a linear axis testbed was designed to allow testing of the new method against a commercial laser-based system. Various degradation patterns can be experimentally simulated by simple substitution of the bearing balls with balls of smaller or greater diameter.

Future tests will reveal the effectiveness of the new sensor-based method. Once the method is verified for diagnostics of linear axes, further tests may show the value of certain metrics for prognostic purposes to estimate the RUL. If the data collection and analysis are integrated within a machine controller, the process may seem to be seamless. Automated diagnostics and prognostics of linear axes can be used to help optimize maintenance and ultimately part quality. Therefore, the method is expected to generate a net positive with respect to decreased production losses for a machine tool.

## Figures and Tables

**Table 1:** Properties of sensors used in sensor box.

Sensor	Bandwidth^a^	Noise
Accelerometer	0.02 Hz to 1700 Hz	2.9 (μm/s^2^)/√Hz at 1 Hz to 0.4 (μm/s^2^)/√Hz at 1 kHz
Inclinometer	0 Hz to 2 Hz	2.4 μrad^b^
Rate gyroscope	0 Hz to 200 Hz	0.002 °/s/√Hz

a frequencies correspond to half-power points, also known as 3 dB points

b maximum deviation at 0 Hz

**Table 2:** Tolerances for linear axis errors of vertical machining centers.

Error	Tolerance*
Straightness	20 μm
Angular (Pitch, Yaw, or Roll)	60 μrad

* for axes capable of 1 meter of travel, according to ISO 10791-2 (International Organization for Standardization, 2001)

**Table 3:** Fixed-cycle test for linear axis with a 1-m travel.

Sensor	Measurand
**Axis Speed = 0.02 m/s**	
Rate Gyroscope	Angular errors, 0.1 mm to 2 mm wavelength
Inclinometer	Angular errors, > 10 mm wavelength
Accelerometer	Straightness errors, 0.1 mm to 10 mm wavelength
**Axis Speed = 0.1 m/s**	
Rate Gyroscope	Angular errors, 2 mm to 10 mm wavelength
Accelerometer	Straightness errors, 10 mm to 100 mm wavelength
**Axis Speed = 0.5 m/s**	
Accelerometer	Straightness errors, 100 mm to 10 m wavelength

**Table 4:** Straightness error uncertainties due to sensor noise.

Sensor	Axis Speed^a^	Filter	Expanded Uncertainty^b^	Standard Uncertainty
Accelerometer	Slow	Band-pass (2 Hz, 200 Hz)	0.081 μm	0.015 μm
Accelerometer	Moderate	Band-pass (1 Hz, 10 Hz)	0.14 μm	0.029 μm
Accelerometer	Fast	Low-pass (5 Hz)	0.26 μm	0.055 μm

a ‘Slow’ speed = 0.02 m/s, ‘Moderate’ speed = 0.1 m/s, and ‘Fast’ speed = 0.5 m/s

b defines an interval estimated to have a level of confidence of 99.8 percent

**Table 5:** Angular error uncertainties due to sensor noise.

Sensor	Axis Speed^a^	Filter	Expanded Uncertainty^b^	Standard Uncertainty
Inclinometer	Slow	Low-pass (2 Hz)	2.4 μrad	1.4 μrad^c^
Rate gyroscope	Moderate	Band-pass (10 Hz, 50 Hz)	6.2 μrad	1.2 μrad
Rate gyroscope	Slow	Band-pass (10 Hz, 200 Hz)	7.3 μrad	1.3 μrad

a ‘Slow’ speed = 0.02 m/s, ‘Moderate’ speed = 0.1 m/s, and ‘Fast’ speed = 0.5 m/s

b defines an interval estimated to have a level of confidence of 99.8 percent

c based on an assumed uniform distribution (NIST/SEMATECH, 2014)

**Table 6:** Uncertainties of sensor-based method.

Error	Runs for Averaging	Expanded Uncertainty^a^,^b^	Standard Uncertainty^a^
Straightness	1	5.6 μm	0.97 μm
Straightness	5	4.1 μm	0.70 μm
Straightness	10	4.0 μm	0.65 μm
Angular	1	12.8 μrad	2.3 μrad
Angular	5	9.0 μrad	1.4 μrad
Angular	10	8.7 μrad	1.3 μrad

a for 100 simulations with different randomly-generated errors over a 1-m travel

b defines an interval estimated to have a level of confidence of 99 percent

## References

[R1] Altintas Y, Verl A, Brecher C, Uriarte L, Pritschow G (2011). Machine tool feed drives. CIRP Annals - Manufacturing Technology.

[R2] Biehl S, Staufenbiel S, Recknagel S, Denkena B, Bertram O (2012). Thin film sensors for condition monitoring in ball screw drives.

[R3] Ehrmann C, Herder S (2013). Integrated diagnostic and preload control for ball screw drives by means of self-sensing actuators. www.scientific.net/AMR.769.271.

[R4] Feng G-H, Pan Y-L (2012). Investigation of ball screw preload variation based on dynamic modeling of a preload adjustable feed-drive system and spectrum analysis of ball-nuts sensed vibration signals. International Journal of Machine Tools and Manufacture.

[R5] Garinei A, Marsili R (2012). A new diagnostic technique for ball screw actuators. Measurement: Journal of the International Measurement Confederation.

[R6] Huang B, Gao H, Xu M, Wu X, Zhao M, Guo L (2010). Life prediction of CNC linear rolling guide based on DFNN performance degradation model.

[R7] International Organization for Standardization (2001). ISO 10791-2 - test conditions for machining centres part 2: Geometric tests for machines with vertical spindle or universal heads with vertical primary rotary axis (vertical Z-axis).

[R8] International Organization for Standardization (2012). ISO 230-1 - test code for machine tools part 1: Geometric accuracy of machines operating under no-load or quasi-static conditions.

[R9] International Organization for Standardization (2014). ISO 230-2 - test code for machine tools part 2: Determination of accuracy and repeatability of positioning of numerically controlled axes.

[R10] Khan AW, Chen W (2009). Calibration of CNC milling machine by direct method.

[R11] Li Y, Wang X, Lin J, Shi S (2014). A wavelet bicoherence-based quadratic nonlinearity feature for translational axis condition monitoring. Sensors.

[R12] Liao L, Lee J (2009). A novel method for machine performance degradation assessment based on fixed cycle features test. Journal of Sound and Vibration.

[R13] Liao L, Pavel R (2012). Machine tool feed axis health monitoring using plug-and-prognose technology.

[R14] Möhring H-C, Bertram O (2012). Integrated autonomous monitoring of ball screw drives. CIRP Annals - Manufacturing Technology.

[R15] NIST/SEMATECH (2014). E-handbook of statistical methods.

[R16] Ouafi AE, Barka N (2013). Accuracy enhancement of CNC multi-axis machine tools through an on-line error identification and compensation strategy. www.scientific.net/AMR.718-720.1388.

[R17] Plapper V, Weck M (2001). Sensorless machine tool condition monitoring based on open NCs.

[R18] Shi R, Guo Z, Song Z, Yan J (2012). Resarch of mechanical components' performance degradation based on dynamic fuzzy neural network.

[R19] Teti R, Jemielniak K, O’Donnell G, Dornfeld D (2010). Advanced monitoring of machining operations. CIRP Annals - Manufacturing Technology.

[R20] Uhlmann E, Geisert C, Hohwieler E (2008). Monitoring of slowly progressing deterioration of computer numerical control machine axes. Proceedings of the Institution of Mechanical Engineers, Part B: Journal of Engineering Manufacture.

[R21] Verl A, Heisel U, Walther M, Maier D (2009). Sensorless automated condition monitoring for the control of the predictive maintenance of machine tools. CIRP Annals - Manufacturing Technology.

[R22] Zhou Y, Mei X, Zhang Y, Jiang G, Sun N (2009). Current-based feed axis condition monitoring and fault diagnosis.

[R23] Zhou Y, Tao T, Mei X, Jiang G, Sun N (2011). Feed-axis gearbox condition monitoring using built-in position sensors and eemd method. Kidlington, Oxfordshire OX5 1GB.

[R24] Zhou Y, Xu H, Liu J, Zhang Y (2014). On-line backlash-based feed-axis wear condition monitoring technology.

